# Supplementary material for: Epistaxis and thrombocytopenia as major presentations of louse borne relapsing fever: Hospital-based study
Source: PLoS One. 2022 Dec 30;17(12):e0279721. doi: 10.1371/journal.pone.0279721 (PMC9803200; doi:10.1371/journal.pone.0279721)
Supplement: Attachment — Submitted filename: Response to Reviewers.docx [file pone.0279721.s001.docx]

# Response to Academic Editor/Journal Requirements

1. Please provide additional details regarding participant consent. In the ethics statement in the Methods and online submission information, please ensure that you have specified what type you obtained (for instance, written or verbal, and if verbal, how it was documented and witnessed). If your study included minors, state whether you obtained consent from parents or guardians. If the need for consent was waived by the ethics committee, please include this information.

- Thank you; we have included the information.

2. We note you have included a table to which you do not refer in the text of your manuscript. Please ensure that you refer to Table 2 in your text; if accepted, production will need this reference to link the reader to the Table.

- Thank you; we have updated it accordingly.

# Response to Reviewers

1. Lines 59-63: This reviewer suggests adding a topic sentence, so the paragraph is not a two-sentence paragraph. For example, the authors could add the following topic sentence, “There are several factors that promote LBRF outbreaks.”

- Response: comment accepted and edited as per the suggestion with a track change in the manuscript.

2. Lines 64-79. These two paragraphs could be combined into one.

- Response: We separated these paragraphs because the first paragraph (line 64-69) focuses on clinical presentation while the second (line 70-69) tries to address diagnostics of LBRF. Merging them may also make the paragraph too long.

3. Lines 74-75. The sentence needs editing. It is written, “Therefore, a quantitative buffy coat (QBC) technique has been reported a sensitive…” This reviewer thinks the authors mean “as a sensitive” I underlined “as” to point out that I believe it is a missing word.

- Response: Thank you; updated as per the suggestion.

4. Lines 75-77. This sentence should be reworded. In particular, the “100% sensitivity and specificity.” Context is needed. For example, when B. recurrentis has been cleared by the host antibody response, PCR will not detect spirochete DNA in the blood. Did the studies in reference 2 and 9 demonstrate detection of Borrelia DNA between spirochetemic episodes?

- Response: Thank you for the comment. We have added the following statement to address the reviewer’s comments “However, PCR detects the infection only at the febrile stage and not during the incubation period prior to the onset of clinical symptoms”. We have also cited the source for the added point.

5. Line 118. The meaning of BF should be written out the first place it appears in the manuscript.

- Response: Thank you, we have updated in the manuscript as ‘blood film (BF)’.

6. Lines 138-140. The sentence beginning with “Chest X-ray…” could be broken up into two sentences. The second sentence should start at, “Findings consistent…”

- Response: We have edited as per the comment.

7. Lines 161-162. This sentence reads awkwardly and needs editing.

- Response: The sentence has been revised to address the comments

8. This reviewer does not think figure 1 is needed.

- Response: We would like to thank the reviewer for the suggestion. However, we prefer to keep the figure as it may give readers a better understanding of what has been briefly stated in the text.

9. Lines 168-170. As written, this is not a sentence. Please correct.

- Response: Thank you, edited as ‘Doxycycline 100 mg twice daily, paracetamol 1 gm at presentation, and intravenous fluid were given for all patients.’

10. Lines 170-172. This sentence is difficult to understand. Please correct.

- Response: This part has been revised in the new version to address the reviewer’s concerns.

11. Line 173. “due their unavailability” is incorrectly written. Also, “their” is not the correct word to use. This sentence may not be needed and could be omitted from the manuscript.

- Response: Omitted as per the suggestion.

12. Line 174. For more clarity, the sentence “All patients had smooth in hospital course after commencement of treatment….” could be written, “All patients recovered after the commencement of treatment….”

- Response: Edited as per the suggestion.

13. Line 177-179. For simplicity, the sentence could be written, “All the patients and their known contacts were deloused.” Also, this sentence should be the topic sentence for the following paragraph.

- Response: Edited as per the comment.

14. Line 186. “Louse” does not need to be capitalized.

- Response: Edited as per the comment

15. Line 205. There needs to be a comma after malaria.

- Response: Edited as per the comment

16. Lines 203-206. This reviewer suggests deleting the words “which are causing occasional outbreaks in the country now”

- Response: Deleted as per the comment

17. Line 234. “thus” should be changed to “which”

- Response: Edited as per the comment

18. Line 243. A comma is needed before “which”.

- Response: Edited as per the comment

19. Line 251. For clarity, a period should be placed after “accordingly” and the next sentence should read, “One patient with known ….”

- Response: Edited as per the comment

20. Line 271. “Besides” should be deleted.

- Response: Deleted as per the comment

21. Lines 277-280. “susception” is confusing. Please edit this sentence. Also, in line 279, “bleeding and thrombocytopenia as important presentations….” could be reworded “We reported that bleeding and thrombocytopenia are important presentations and complications.” Overall, the sentences in lines 277-283 are difficult to understand. Editing is needed.

- Response: The term “susception” is updated as ‘suspicion’
- All cases were not confirmed by laboratory. Among the 36 patients, only 14 patients were confirmed by blood film detection. However, by considering the patients high risk setting (prison), the rest cases were considered as probable case due to high suspicion for the case despite it was not confirmed by blood film.
- Hence, this study highlighted the importance of high index of suspicion of cases for the diagnosis and treatment of LBRF in “high-risk settings
